# Supplementary material for: Colorectal Air–Liquid Interface Organoids Preserve Tumour-Immune Architecture and Reveal Local Treg Expansion After PD-1 Blockade
Source: Cancers (Basel). 2025 Dec 30;18(1):132. doi: 10.3390/cancers18010132 (PMC12784706; doi:10.3390/cancers18010132)
Supplement: Supplementary file 1 [file cancers-18-00132-s001.zip › cancers-4051183-supplementary.pdf]

Supplemental information

Colorectal Air–Liquid Interface Organoids Preserve Tumour-Immune Architecture and Reveal Local Treg Expansion After PD-1 Blockade

Supplementary Tables

Supplementary Table S1. Clinicopathological characteristics of colorectal cancer patients from whom biopsies were obtained to generate Matrigel and ALI-based organoid cultures (N = 33).

Patient ID #	Sex	Age	Diagnosis	TNM	Differentiation	Stage	Location	Analysis in this study
#1	F	59	Adenocarcinoma	pT4a pN0 R0	G2	IIB	Ascending	Immune cell composition (immunofluorescence, spectral flow cytometry). Soluble immune checkpoint (IC) and cytokine detection assays
#2	M	59	Adenocarcinoma	pT1 pN0 R0	G2	I	Sigma	
#3	F	88	Mucinous adenocarcinoma	pT3 N0 R0	Not available	IIA	Ascending	
#4	F	87	Adenocarcinoma	pT3 N0 R0	G2	IIA	Ascending	
#5	F	57	Adenocarcinoma	pT3 pN1a R0	G2	IIIA	Sigma	
#6	F	75	Adenocarcinoma	pT2 pN0 R0	G2	I	Rectum	
#7	M	86	Adenocarcinoma	pT4a pN1b R1	G2	IIIB	Cecum	
#8	F	91	Mucinous adenocarcinoma	pT4a pN0 R0	Not available	IIB	Ascending	Whole Exome Sequencing (WES)
#9	F	57	Adenocarcinoma	pT3 pN1a R0	G2	IIIA	Sigma	
#10	M	85	Micropapillary carcinoma	pT3 pN0 R0	G3	IIA	Sigma	
#11	M	76	Adenocarcinoma	Not available	Not available	Not available	Cecum	
#12	F	79	Adenocarcinoma	pT4a N0 R2	G2	IIB	Sigma	
#13	M	69	Adenocarcinoma	pT3 pN2a R0	G2	IIIB	Ascending	
#14	F	79	Mucinous adenocarcinoma	pT3 N0 R0	Not available	IIA	Sigma	
#15	M	82	Adenocarcinoma	pT4a pN1a R0	G2	IIIB	Ascending	5-FU assays
#16	M	72	Adenocarcinoma	pT2 N0 R0	G1-2	I	Ascending	
#17	F	79	Mucinous adenocarcinoma	pT3 N0 R0	Not available	IIA	Sigma	
#18	F	57	Adenocarcinoma	pT4a N1b R0	G2	IIIB	Rectum	
#19	M	39	Adenocarcinoma	pT4 pN2a	G3	IIIC	Sigma	
#20	M	76	Adenocarcinoma	pT3 pN1b R0	G2	IIIB	Ascending	

#21	M	87	Adenocarcinoma	pT3 N0 R0	G3	IIA	Cecum	
#22	M	85	Adenocarcinoma	pT2 pN0 R0	G2	I	Sigma	
#23	M	66	Adenocarcinoma	pT3 pN1c R0	G2	IIIB	Descending	
#24	M	65	Adenocarcinoma	pT1 pN0 R0	G2	I	Sigma	
#25	M	64	Adenocarcinoma	pT4a pN1b R0	G2	IIIB	Ascending	Nivolumab assays
#26	M	65	Micropapillary carcinoma	pT3 pN2a R0	G3	IIIB	Descending	
#27	M	67	Adenocarcinoma	pT1 pN0 R0	Not available	I	Rectum	
#28	M	52	Adenocarcinoma	pT1 pN0 R0	Not available	I	Sigma	
#29	F	79	Adenocarcinoma	pT4b pN1b R2	G2	IIIC	Sigma	
#30	F	62	Mucinous adenocarcinoma	pT4a pN1b	Not available	IIIB	Ascending	
#31	M	76	Adenocarcinoma	pT3 pN0 R0	G2	IIA	Ascending	
#32	M	66	Adenocarcinoma	pT3 pN2a R0	G3	IIIB	Ascending	Comparative coculture and ALI-PDO study
#33	M	65	Adenocarcinoma	pT4a pN0 R0	G2	IIB	Descending	

F: Female, M: Male, TNM staging system: the size of the primary tumour (T), the extent to which it has spread to nearby lymph nodes (N), and whether it has spread to other parts of the body (M)

Supplementary Table S2. List of antibodies used for the analysis of immune infiltrate populations in PDOs by spectral flow cytometry.

ANTIBODIES	SOURCE	IDENTIFIER
LIVE/DEAD BLUE	ThermoFisher	L34957
Brilliant Violet 421 anti-human CD197 (CCR7)	Biolegend	Cat#353208, RRID:AB_11203894
BUV563 Mouse anti-Human CD195 (CCR5)	BD	Cat# 741401, RRID:AB_2870893
PE/Cyanine7 anti-human CD183 (CXCR3)	Biolegend	Cat# 353720, RRID:AB_11219383
BV750 anti-human CXCR5 (CD185)	BD	Cat# 747111, RRID:AB_2871862
PerCP-eFluor 710 anti-human TCRγδ	ThermoFisher	Cat # 46-9959-42, RRID AB_2573926
PerCP/Cyanine5.5 anti-human CD11b	Biolegend	Cat# 301328, RRID:AB_10933428
PE/Cy5 anti-human CD95	Biolegend	Cat# 311134; RRID:AB_2566349
PE-Alexa Fluor™ 700 CD25 Monoclonal Antibody	ThermoFisher	Cat# MHCD2524, RRID:AB_2539740
APC anti-human CD27	Biolegend	Cat# 356410, RRID:AB_2561957
Alexa Fluor(R) 647 anti-human CD1c	Biolegend	Cat# 331510, RRID:AB_1186032
eFluor™ 450 anti-human CD163	ThermoFisher	Cat # 48-1637-42, RRID:AB_2815180

Brilliant Violet 711 anti-human CD206	Biolegend	Cat # 321136, RRID: AB_2687199
Brilliant Violet 510™ anti-human CD3	Biolegend	Cat# No. 317331, RRID: AB_2561376
Pacific Orange anti-Human CD20	ThermoFisher	Cat# MHCD2030, RRID:AB_10375578
BV605 Mouse Anti-Human IgG	BD	Cat# 563246, RRID: AB_2738092
Brilliant Violet 785anti-human CD279 (PD-1)	Biolegend	Cat# 329929, RRID:AB_11218984
APC-R700 Mouse Anti-Human CD127	BD	Cat# 565185, RRID:AB_2739099
BUV661Anti-human CD11c	BD	Cat# 612967, RRID:AB_2870241
BUV737 anti-human CD56	BD	Cat# 612766, RRID:AB_2813880
Brilliant Violet 570 anti-human IgM	Biolegend	Cat# 314517, RRID:AB_10913816
Brilliant Violet 650 anti-human CD28	Biolegend	Cat# 302946, RRID:AB_2616855
BV570 Anti-human CD4	CytekBiosciences	Cat# 300534; RRID: AB_2563791
BB515 Anti-human CD141	BD	Cat# 566017; RRID:AB_2739462
Spark Blue 550 anti-human CD14	Biolegend	Cat# 367148, RRID:AB_2832724
PerCP anti-human CD45	Biolegend	Cat# 368506, RRID:AB_2566358
PE/Cy5 Anti-human CD95	Biolegend	Cat# 305610, RRID:AB_314548
APC/Fire™ 750 anti-human HLA-DR	Biolegend	Cat# 307658, RRID:AB_2572101
BUV395 Mouse Anti-Human CD45RA	BD	Cat# 740315, RRID:AB_2740052
BUV805 Mouse Anti-Human CD8	BD	Cat# 612889, RRID:AB_2833078
Super Bright™ 436 anti-human CD123	ThermoFisher	Cat# 62-1239-42, RRID:AB_2662727
APC/Fire 810 anti-human CD38	Biolegend	Cat# 303550, RRID:AB_2860784
BUV496 Mouse Anti-Human CD16	BD	Cat# 612944, RRID:AB_2870224
FITC anti-human CD57	Biolegend	Cat# 359604, RRID:AB_2562387
BV480 Mouse Anti-Human IgD	BD	Cat#566138; RRID: AB_2739536
BUV615 Anti-Human CD62L	BD	Cat# 751364, RRID:AB_2875371

Supplementary Table S3. Mean and standard deviation of sample read depth.

Sample	Average_Coverage
#8_Tissue	57.77
#8_Matrigel	55.61
#8_ALI	51.5
#9_Tissue	42.94
#9_Matrigel	43.32
#9_ALI	45.4
#10_Tissue	63.54
#10_Matrigel	56.91
#10_ALI	50.87
#11_Tissue	48.26
#11_Matrigel	42.02
#11_ALI	38.39
#12_Tissue	61.52
#12_Matrigel	58.16

#12_ALI	58.86		
#13_Tissue	46.12		
#13_Matrigel	45.51		
#13_ALI	50.24		
#14_Tissue	48.83		
#14_Matrigel	53.85		
#14_ALI	49.07		
	Tissue	Matrigel	ALI
Mean depth of samples	52.4	50.6	48.6
Standard deviation depth of samples	7.6	6.4	6.0

Supplementary Table S4. DNA reads and mutations sample quality data: raw DNA starting read pairs, reads pairs remaining after adapter trimming, reads and percentage mapped in proper read pairs to the hg38 human genome (no alternative contigs), variants with respect to the reference genome and average coverage in WES gene panel regions.

	#8_Tissue	#8_Matrigel	#8_ALI	#9_Tissue	#9_Matrigel	#9_ALI	#10_Tissue	#10_Matrigel	#10_ALI
Starting read pairs	25351627	26786215	26350190	22485706	21929401	21974667	25301294	29600432	30871235
Trimmed read pairs	25350920	26785558	26349202	22485052	21928389	21972942	25297611	29598204	30869669
Reads mapped in proper pairs	46514744	52703070	52037296	44045442	43284608	43349962	49239074	57206544	60909852
% of reads mapped in proper pairs	91,74	98,38	98,75	97,94	98,7	98,64	97,32	96,64	98,66
Variants	3477	3891	3682	2250	2578	2222	2324	2502	2410
Average coverage	51,5	55,61	57,77	45,4	43,32	42,94	50,87	56,91	63,54

	#11_Tissue	#11_Matrigel	#11_ALI	#12_Tissue	#12_Matrigel	#12_ALI	#13_Tissue	#13_Matrigel	#13_ALI
Starting read pairs	20987697	22028433	23431942	26905613	27859026	28752857	23239987	23562764	21966066
Trimmed read pairs	20987367	22027485	23430220	26904864	27857368	28751740	23239710	23561415	21965812
Reads mapped in proper pairs	40768742	43264714	46361516	53209492	54901988	56882936	45522942	46212268	43569880
% of reads mapped in proper pairs	97,13	98,21	98,94	98,88	98,54	98,92	97,94	98,07	99,18
Variants	2287	2994	2262	2967	3892	3066	2282	6184	2308
Average coverage	38,39	42,02	48,26	58,86	58,16	61,52	50,24	45,51	46,12

	#14_Tissue	#14_Matrigel	#14_ALI
Starting read pairs	23787285	26754864	21879652
Trimmed read pairs	23785621	26746023	21879051
Reads mapped in proper pairs	46103254	52557472	43111712
% of reads mapped in proper pairs	96,91	98,25	98,52
Variants	5119	7865	5193
Average coverage	49,07	53,85	48,83

	Tissue	Matrigel	ALI
Mean sample mutations	2878,878884	3920,02813	2826,467352
Standard deviation sample mutation	1099,446809	2020,345409	1060,644458

Supplementary Figures

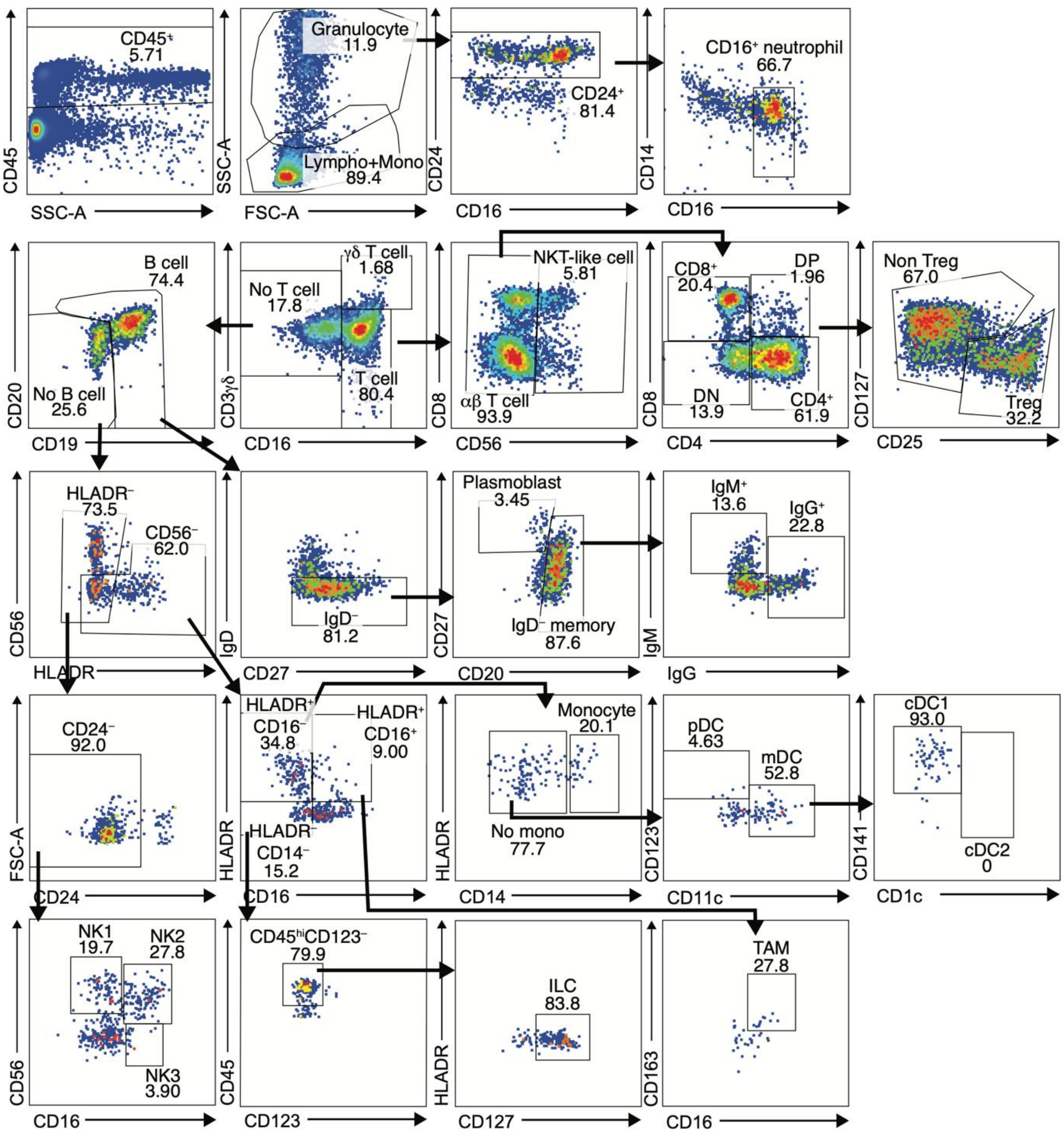

Supplementary Figure S1. **Cell lineage gating strategy for the characterization of the immune infiltrate within tissues and ALI-PDOs**.

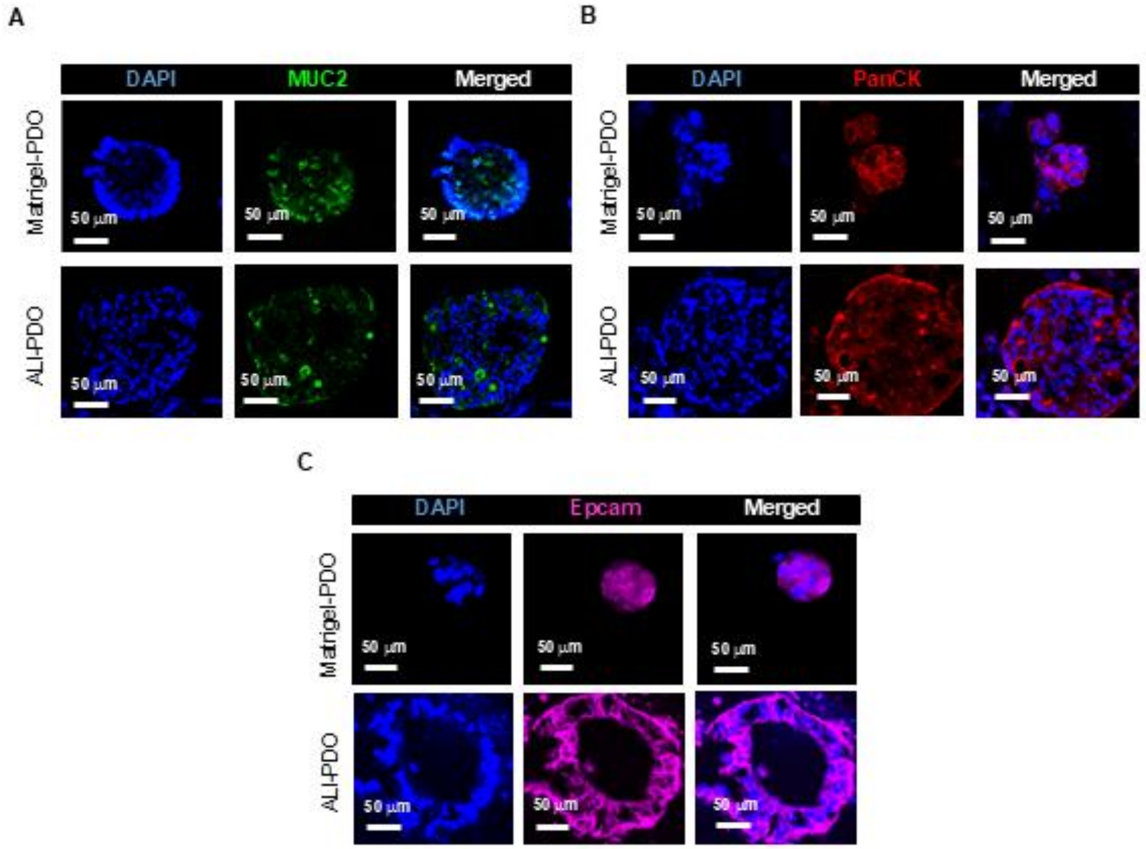

Supplementary Figure S2. Differentiation cell markers in Matrigel and ALI-PDOs. Representative confocal microscopy images of Matrigel and ALI tumour PDOs from **(A)** patient #3 stained for DAPI (blue, nuclear marker), and Mucin-2 (green), (**B**) patient #2 stained for DAPI (blue, nuclear marker), and pan-Cytokeratin (red), and (**C**) patient #1 stained for DAPI (blue, nuclear marker), and Epcam (magenta). Original magnification: 4x; scale bar: 50 µm.

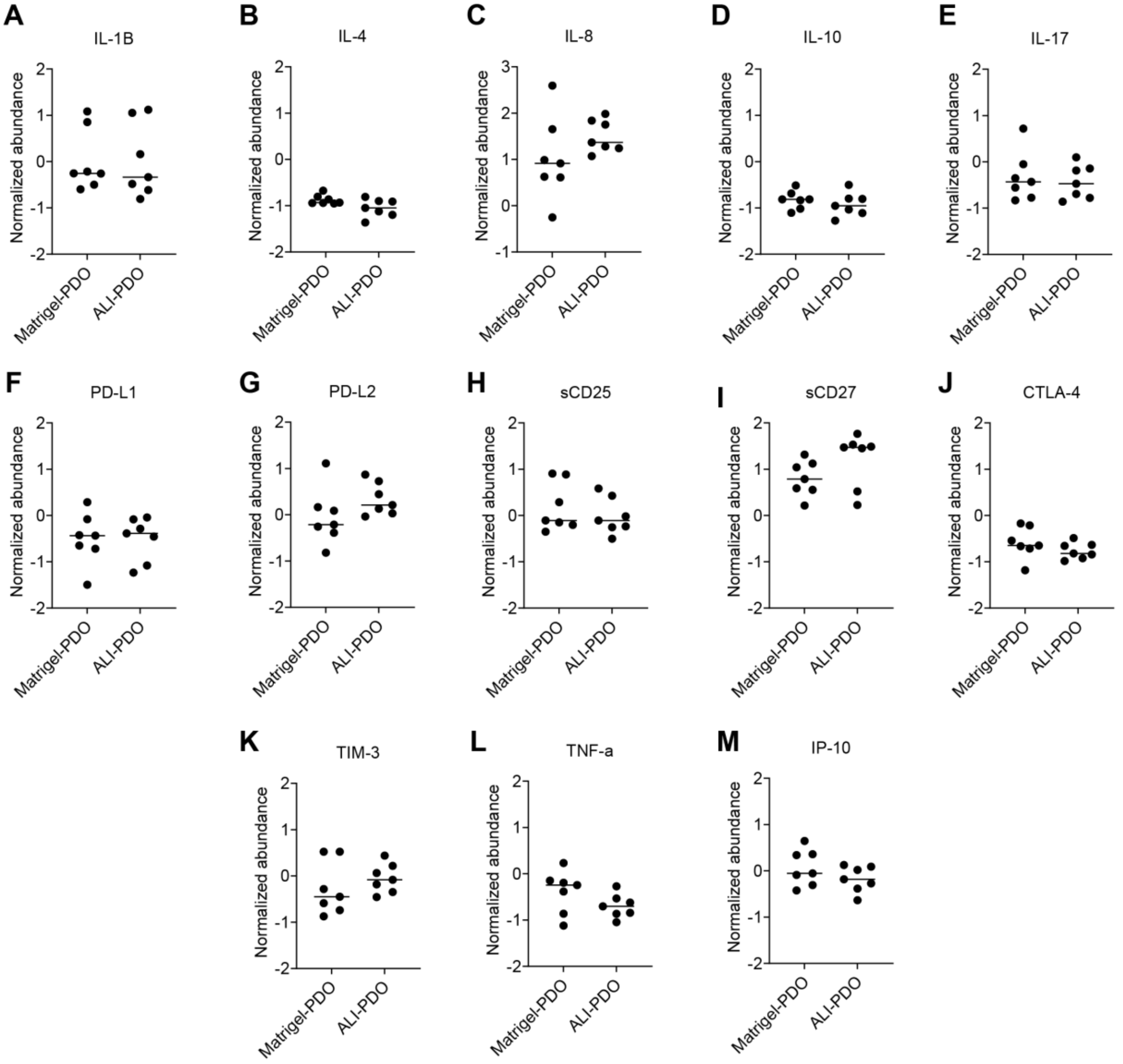

Supplementary Figure S3 Comparative analysis of immune checkpoints and soluble cytokines. (**A-M**) Determination of IL-1b, IL-4, IL-8, IL-10, IL-17, PD-L1, PD-L2, sCD25, sCD27, CTLA-4, TIM-3, TNFa, and IP-10 in the culture supernatants of Matrigel- and ALI-PDOs cultures at day 7, n=7.

Statistical significance was assessed via unpaired Mann-Whitney test (*p < 0.05, **p <0.01)

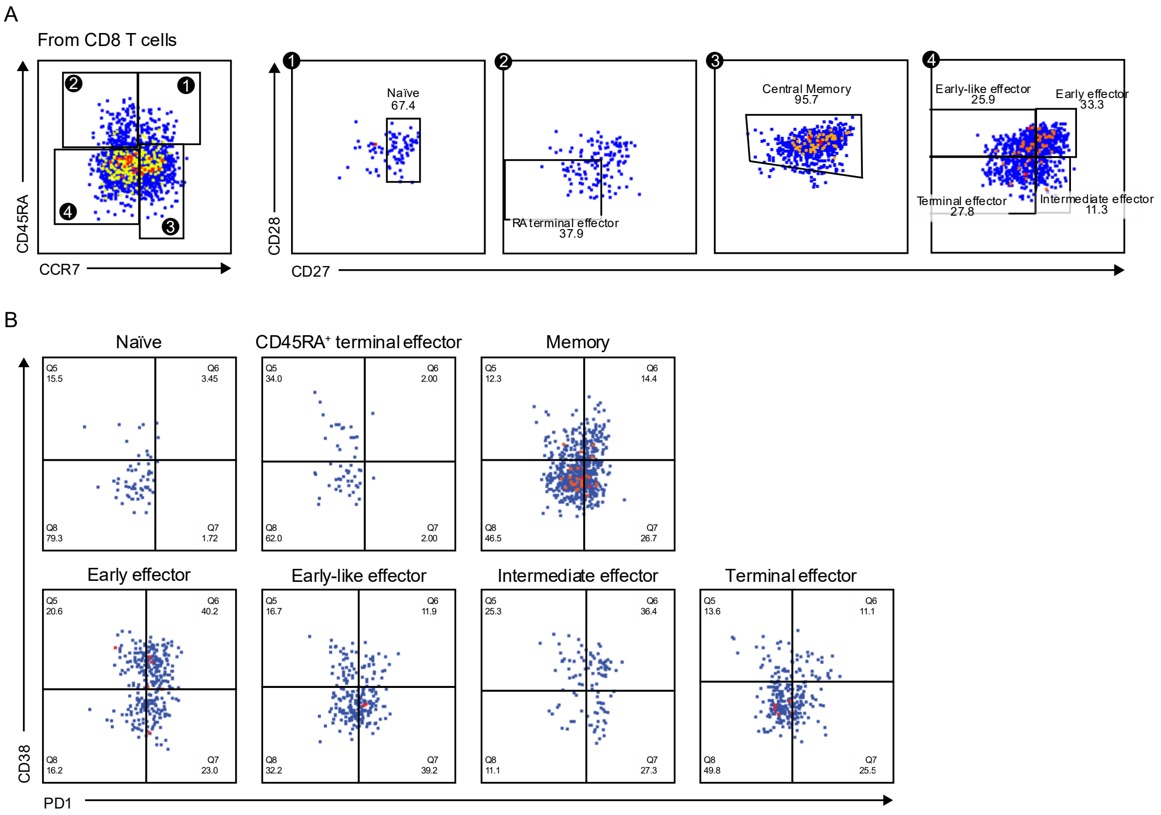

Supplementary Figure S4. Evaluation of exhaustion markers in CD8 T cells subsets from primary tissue and ALI-PDOs. (**A**) Representative gating strategy of differentiation stages of tumour-infiltrating CD8 T cells. (**B**) Representative dot plots of CD38 and PD-1 expression in CD8 T cell subsets.

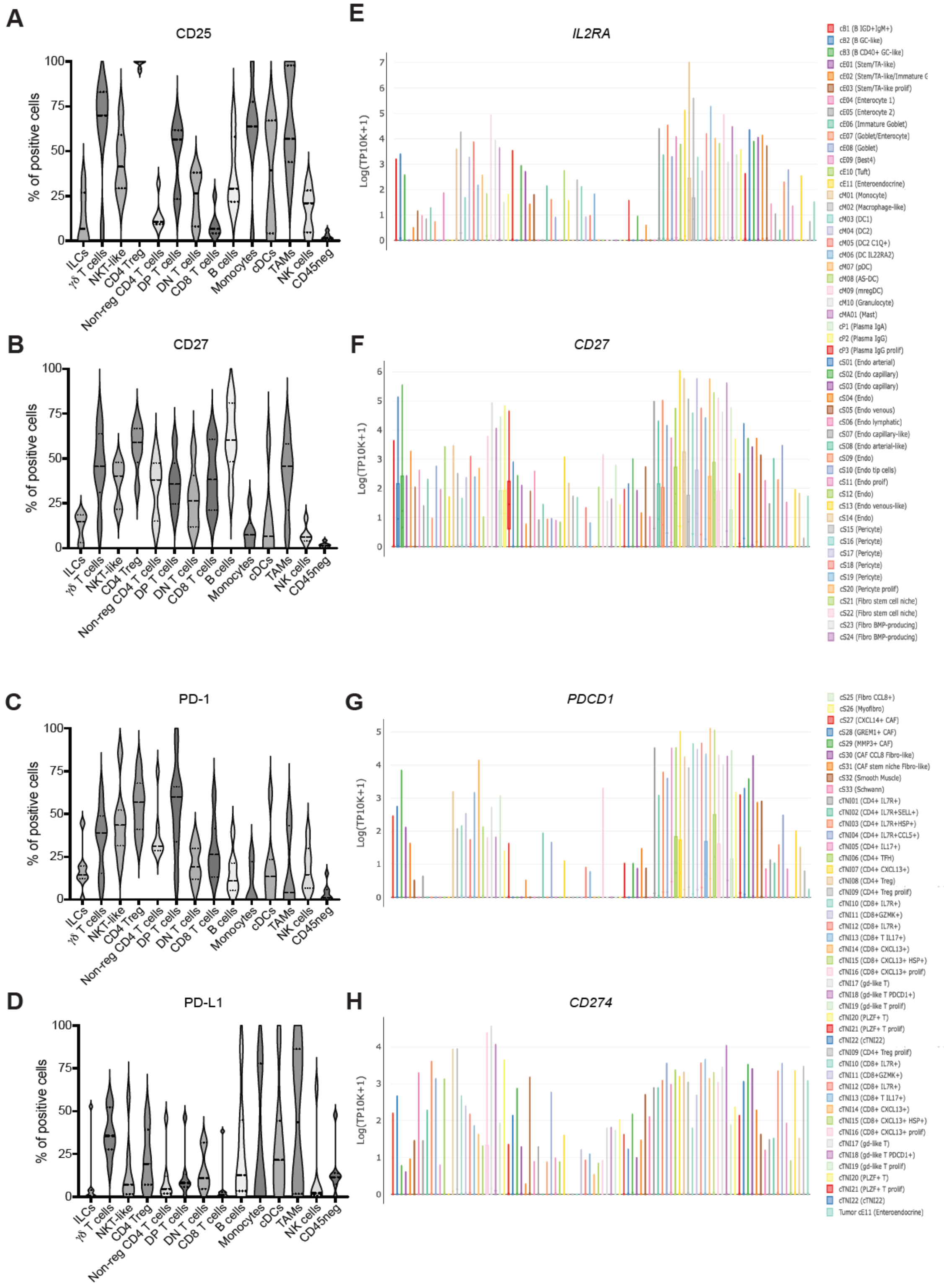

Supplementary Figure S5. Membrane-bound immune checkpoint expression in ALI-PDOs and validation using single-cell transcriptomics. (**A–D**) Flow cytometry analysis of membrane-bound levels of CD25, CD27, PD-1, and PD-L1 across immune and non-immune compartments. CD25, CD27, and PD-1 were significantly enriched in CD45^{+} cells, particularly lymphocytes, whereas PD-L1 was predominantly expressed on myeloid cells, with a substantial fraction of CD45^{-} cells also positive. (**E–H**) Corresponding

single-cell RNA sequencing data from the Human Colon Cancer Atlas (c295), showing expression of IL2RA, CD27, PDCD1, and CD274 across immune and non-immune cell populations.

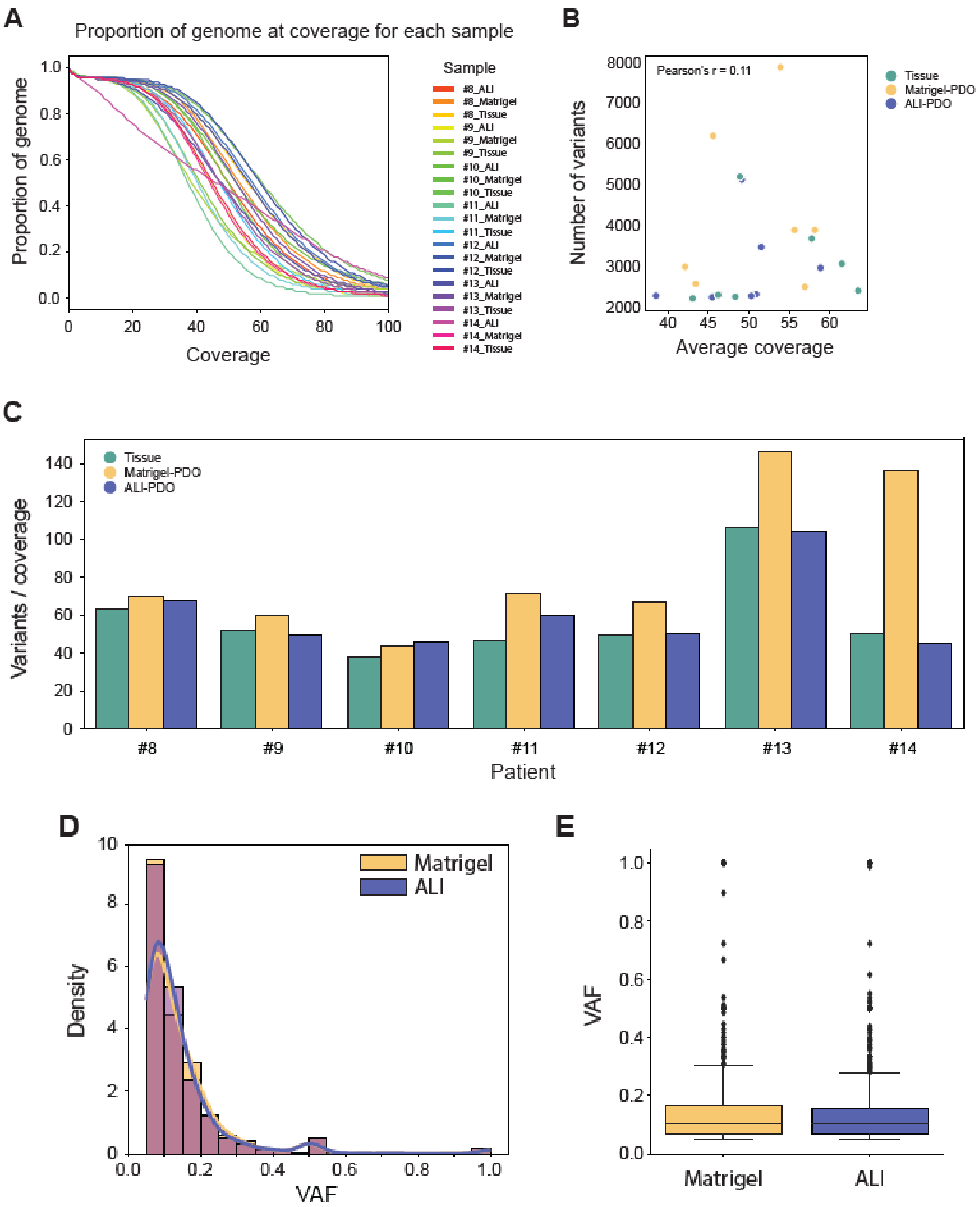

Supplementary Figure S6. **Whole exome sequencing statistics.** (**A**) Read coverage plot with proportion of

the WES gene panel covered for primary tumours, Matrigel- and ALI-PDOs samples. (**B**) Average coverage with number of variants and Pearson Correlation coefficient, sample type (primary tumours, Matrigel- or ALI-PDOs) shown in different colours. (**C**) Sample mutations per average coverage grouped per patient, sample type shown in different colours. (**D**) Distribution of variant allele frequencies for variants present in tissue but absent in Matrigel- or ALI-PDOs. (**E**) Boxplot distribution of variant allele frequencies for variants present in tissue but absent in Matrigel- or ALI-PDOs.
